# Supplementary material for: Refining the Role of Pyruvate Dehydrogenase Kinases in Glioblastoma Development
Source: Cancers (Basel). 2022 Aug 2;14(15):3769. doi: 10.3390/cancers14153769 (PMC9367285; doi:10.3390/cancers14153769)

Figure 2B

PDHK1 (45 kDa)

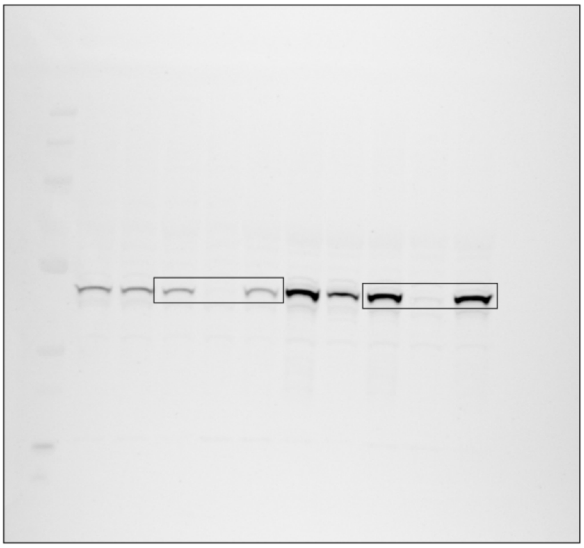

Vinculin (117 kDa)

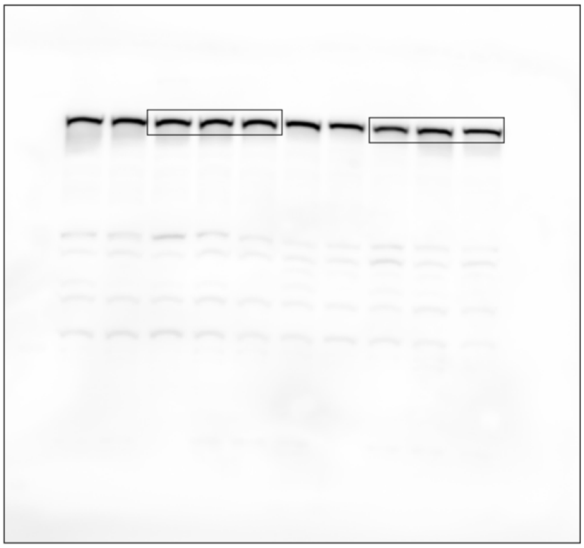

PDHK2 (45 kDa)

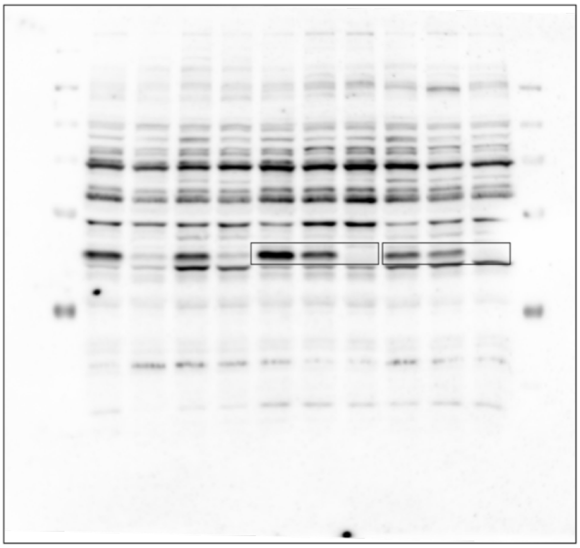

Vinculin (117 kDa)

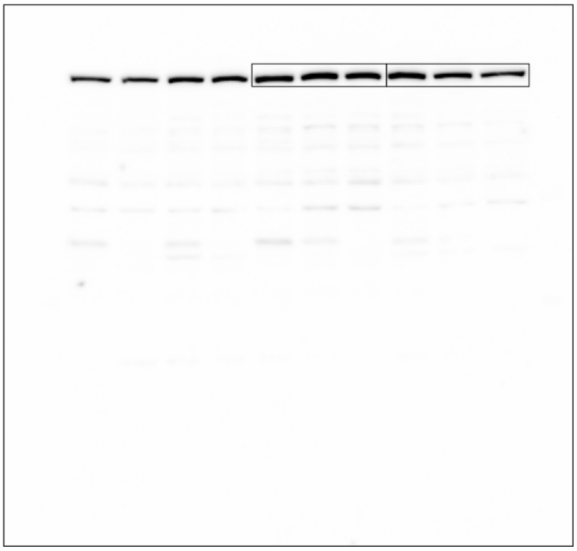

Figure 2C

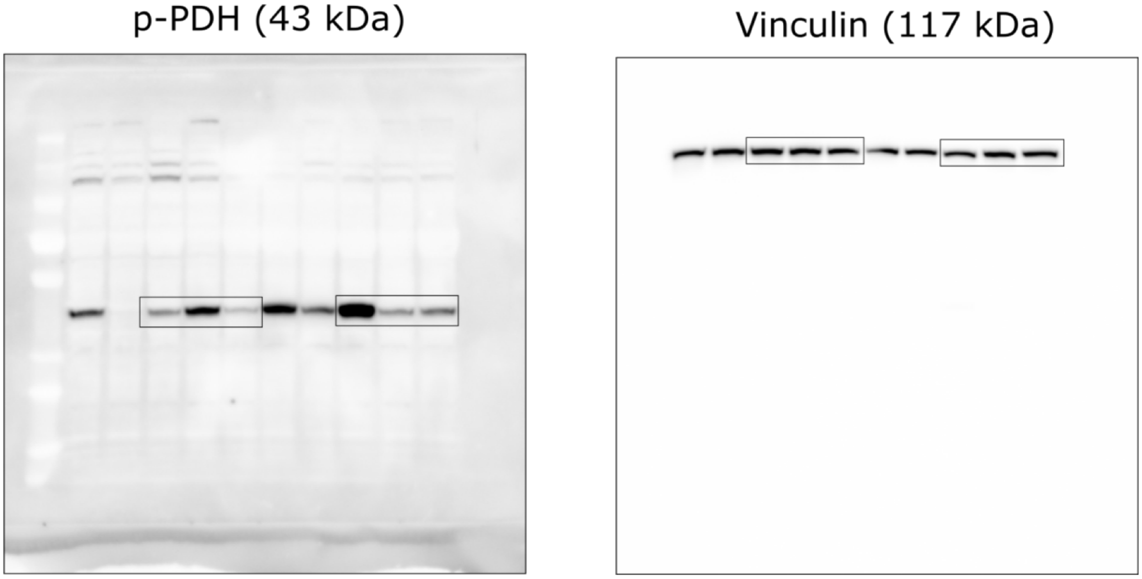

Figure 2D

PDHK1 (45 kDa)

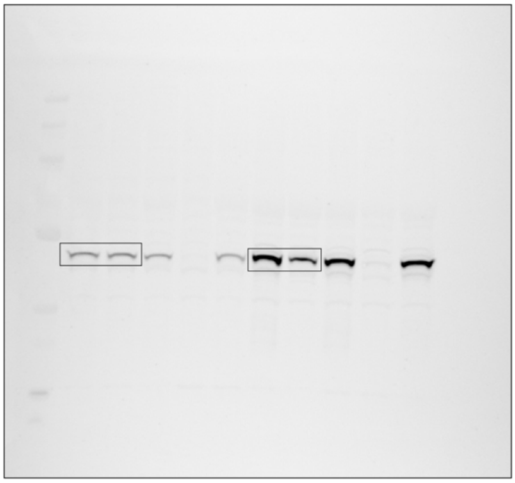

PDHK2 (45 kDa)

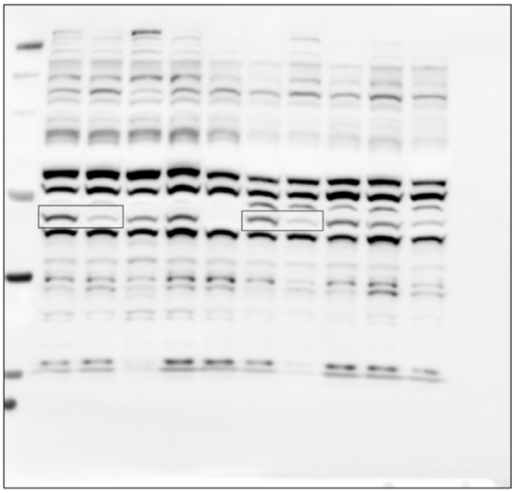

Vinculin (117 kDa)

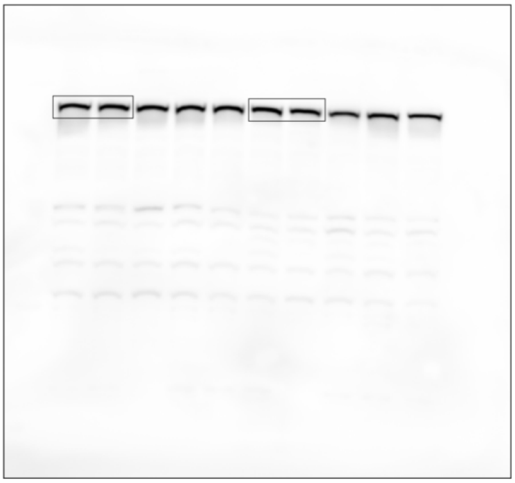

Figure 2E

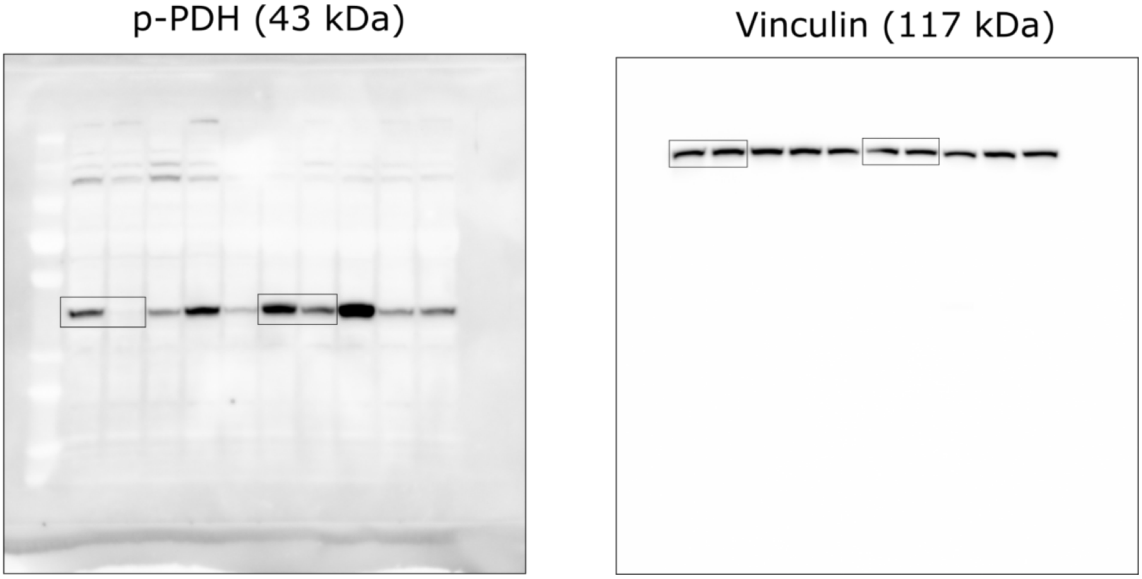

Supplement: Supplementary file 1 [file cancers-14-03769-s001.zip › cancers-1775265-supplementary.pdf]
